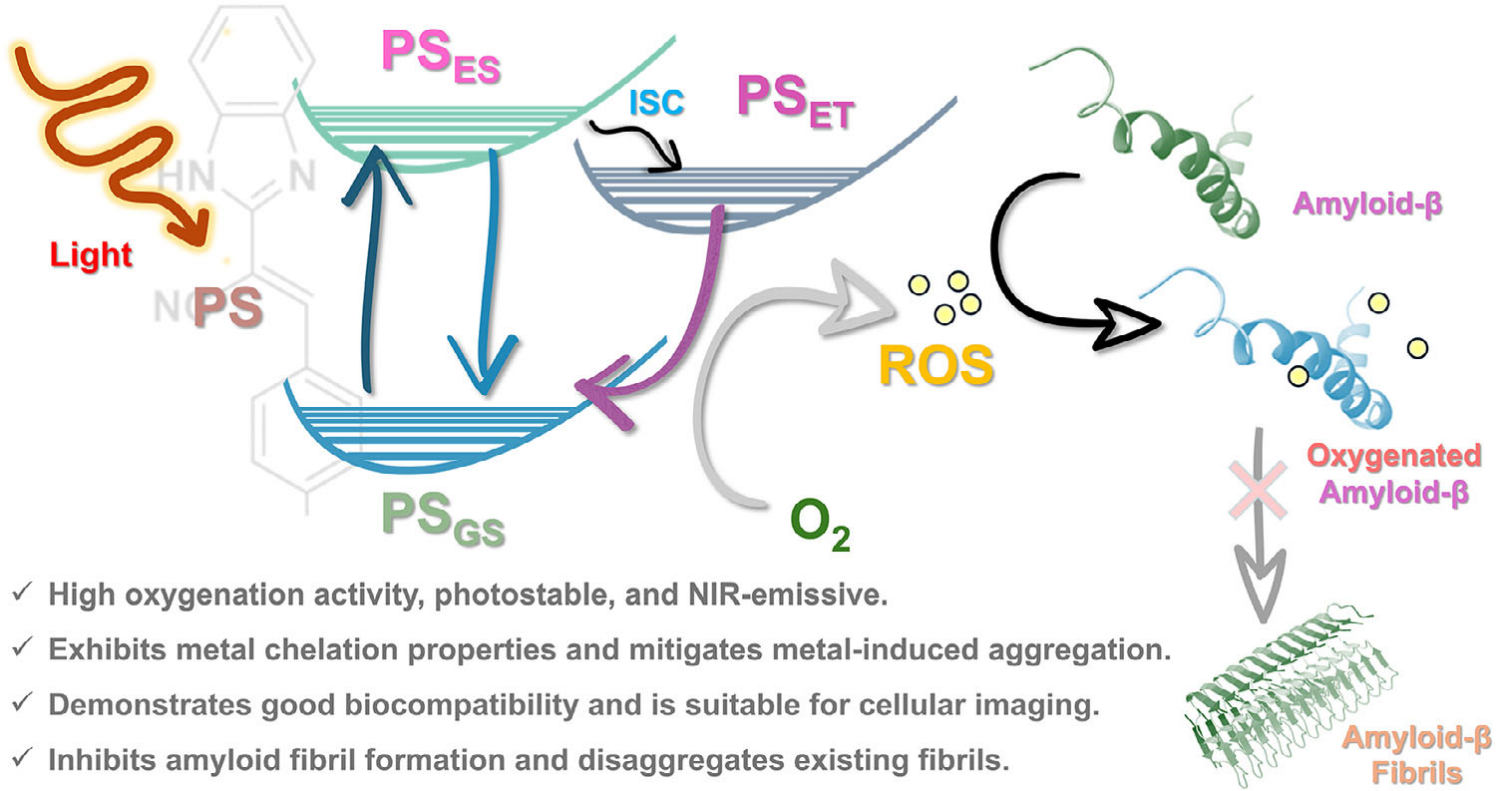# Photoactivable Small Molecule AIE Probes: Exploring Potentials for Alzheimer's Disease Detection and Therapy

**DOI:** 10.1002/alz70855_097195

**Published:** 2025-12-23

**Authors:** Priyam Ghosh

**Affiliations:** ^1^ IIT Guwahati, Guwahati, Assam, India

## Abstract

**Background:**

Alzheimer's disease (AD) is a widespread neurological disorder characterized by cognitive decline and amyloid‐beta (Aβ) plaque buildup, with limited current treatments, underscoring the need for innovative diagnostic and therapeutic strategies.

**Method:**

The selective degradation of amyloids via small molecule‐catalyzed photo‐oxygenation of Aβ has been considered an efficient way to inhibit Aβ aggregation in AD.

**Result:**

This work presents the rational design, synthesis, and evolution of novel multifunctional benzimidazole‐derived photoactivable AIE (aggregation‐induced emission) probes with theranostic potential for AD. Our findings show that benzimidazole‐functionalized probes can potentially interact with Aβ aggregates and reduce Aβ fibril formation, making them therapeutically effective. The probes' capacity to photooxygenate Aβ, block reactive oxygen species (ROS)‐mediated apoptosis, chelate metal ions like Fe(III), good cellular uptake, and mitigate metal‐induced aggregation and oxidative stress demonstrates their promise as effective theranostic agents in AD.

**Conclusion:**

These fluorescent probes' multipotent activity could significantly improve early detection and give a potential therapeutic strategy for slowing the progression of AD. This study opens up new possibilities for creating a multifunctional molecule as a comprehensive diagnostic and therapeutic tool in neurodegenerative disorders.